# What is the value of peer involvement in advancing tobacco harm reduction?

**DOI:** 10.1186/s12954-018-0275-1

**Published:** 2019-01-07

**Authors:** Caitlin Notley, Sharon Cox, Sarah Jakes, Louise Ross

**Affiliations:** 1 Norwich Medical School, University of East Anglia, Norwich Research Park, Norwich, NR4 7TJ UK; 2 Centre for Addictive Behaviours Research, School of Applied Sciences, London South Bank University, 103 Borough Road, London, SE1 0AA UK; 3 New Nicotine Alliance, London, UK; 4 National Centre for Smoking Cessation and Training, London, UK

**Keywords:** Tobacco harm reduction, Vaping, Peer involvement

E-cigarettes are considered a disruptive technology [1], evidencing rapid growth in the financial market and attracting a distinct new customer base of would-be quitters, especially in Great Britain and the USA. Where use is permitted, and regulation is favourable to users [2], e-cigarettes have become the most popular method of smoking cessation [3] and for many are a long-term alternative to smoking [4]. Outside of a medical lens, vaping can be recreational, not just a smoking cessation aid [5]. Simply trying vaping has, for many, had the happy side effect of encouraging smoking cessation through a non-medicalised route. Despite the lack of early research endorsement and the absence of health messaging on e-cigarette use, smokers experimented with them anyway, with many making the switch and quitting smoking completely of their own accord. As the evidence supporting their use as a reduced risk product and a substitute for smoking grew, so too have the endorsements, such that the UK now leads the way in a ‘cautiously permissive’ stance towards the use of e-cigarettes [6, 7].

E-cigarettes are a consumer-led movement. Understanding how and why so many were using vaping products has become an area for research, initially led by consumers, now at the forefront of smoking cessation literature. At the same time, vape shops especially those offering an ‘expert by experience’ contact have become an important source for support [8]. Researchers and stop smoking services began to reach out to vapers. Quitting smoking by vaping may be considered uniquely different from quitting using other available methods of nicotine replacement. In addition to replacing nicotine, vaping replaces many of the behavioural, sensory and social aspects of smoking and a culture and language has developed around it through peer to peer contact and support. Because vaping is so different from other types of tobacco cessation support, for those researchers who have engaged with consumers (and unfortunately this is still not common practice), the assistance offered has been especially valuable in advice around the array of available products, including practical advice, e.g. choice of products, e-liquid flavours, device battery life and real-world patterns of use.

The authors of this article have all actively worked together in shaping active and potential research projects, including narratives on the often overlooked and undervalued pleasures of nicotine [9]. User experience and insight has been essential. Sarah Jakes, a vaper and active advocate, Chair of the New Nicotine Alliance, has been a key trouble shooter. The research team at the University of East Anglia take a social perspective to understand user patterns of e-cigarette use that may support not only smoking cessation, but long-term smoking abstinence. This important qualitative work was initially developed working with Sarah to develop research questions and a funding application. Sarah was fully involved in the CRUK funded ECtra study, from conception, commenting and making changes to the funding application and research materials, advising and assisting with study recruitment, commenting on emergent findings and fully contributing to publications as both an advisor and co-author. She is currently actively involved in feasibility research at London South Bank University, offering e-cigarettes to homeless smokers. Her involvement ranges from device advice through to helping to train homeless support staff with little or no experience with vaping. The advice offered provides expertise which is practical, accessible and reassuring to those undertaking research in the real world.

For researchers, working with peers has required a full consideration of the lived experience of vaping and continually questions the validity and applicability of research findings to the lives of real people.

For vapers without previous research experience, entering the research space is initially a daunting prospect. Communications between researchers can be full of unfamiliar jargon and acronyms, and research concepts may be completely alien. This can lead to feelings of inadequacy and disengagement. To avoid this, it is important to set out the role of peers in research at an early stage, and to be prepared to explain both terminology and methodology in lay terms. This is appropriate communication, rather than a ‘dumbing down’ of the research process. The value of peers in research is their lived experience, not their ability to fully understand complex analytical methodologies. Peers may be able to identify emerging themes that to researchers may be simply ‘lost in the noise’, and further analysis can provide novel avenues for investigation which otherwise might be missed.

In the UK, the peer involvement approach has also extended to stop smoking services, who have increased their enthusiasm for listening to and working with vapers. In Leicester (a city in the Midlands, England), for example, adoption of an action-based research model was influential, where vapers were invited to team meetings and were surveyed post-quit to establish what particularly it was about the vaping experience that meant this quit was more successful than previous attempts (‘I don’t know, it just looked like smoke, and that made me feel happier’). Initially suspicious of e-cigarettes in 2014, the team shared feedback from service users, becoming more confident to be bolder in talking about vaping.

One advisor observed that ‘vapers are becoming stop smoking advisors’. Unlike those who had quit smoking with licensed medication, those who quit with vaping became powerful advocates for switching among their friends and family, sharing their devices and giving encouragement to those still smoking to try vaping. Evidence from stop smoking services in England demonstrates superior quit rates among those who chose a non-licensed product, of between 14 and 20% greater than for prescribable nicotine replacement therapy alone. Currently, we are seeing the involvement of peers shaping smokefree policies by advising on approaches to supporting vaping as a smoking cessation tool.

Further examples of peer involvement in advancing tobacco harm reduction can be seen among a sub-group of smokers who urgently need support: people with poor mental health. In this group, rates of tobacco smoking are much higher than the general population [10]. There are many mental health trusts in England now where the successful use of e-cigarettes has helped inpatients cope with smoke-free policies, encouraging others to see the benefits, including health improvement, ease of maintaining nicotine levels without flouting local policy, financial savings and a tool to maintain a smoke-free life on discharge. The pace of change we see now would not have happened if early adopters had not been heard when they held their vaporiser up and said ‘This does it for me’. Such clarion examples of how peer influence can have an impact on real-world outcomes should be a positive trigger for other research departments to adopt the expertise of those with lived experience.

In future, we hope to see vapers as advocates advancing the research agenda through posing new research questions. The value of peer involvement in tobacco harm reduction is that, through interdisciplinary research, equally valuing the input of ‘experts by experience’ with academic specialisms, we will reach evidence-based answers to important research questions exploring what is essentially a peer-led phenomenon, with unprecedented potential for harm reduction.
